# Supplementary material for: The effect of short-course point-of-care echocardiography training on the performance of medical interns in children
Source: PLoS One. 2022 Dec 15;17(12):e0278173. doi: 10.1371/journal.pone.0278173 (PMC9754179; doi:10.1371/journal.pone.0278173)
Supplement: Attachment — Submitted filename: Response to Reviewers.docx [file pone.0278173.s004.docx]

Dear editor in chief,

We appreciate your great comments, we revised our article based on these comments.

2. Please provide additional details regarding participant consent. In the ethics statement in the Methods and online submission information, please ensure that you have specified (1) whether consent was informed and (2) what type you obtained (for instance, written or verbal, and if verbal, how it was documented and witnessed). If your study included minors, state whether you obtained consent from parents or guardians. If the need for consent was waived by the ethics committee, please include this information.
If you are reporting a retrospective study of medical records or archived samples, please ensure that you have discussed whether all data were fully anonymized before you accessed them and/or whether the IRB or ethics committee waived the requirement for informed consent. If patients provided informed written consent to have data from their medical records used in research, please include this information.

We appreciate your comment. We modified and added the following statement regarding the consent letter. The informed written consent letter was obtained from the parents or guardians before enrollment.

3. You indicated that you had ethical approval for your study. In your Methods section, please ensure you have also stated whether you obtained consent from parents or guardians of the minors included in the study or whether the research ethics committee or IRB specifically waived the need for their consent.

We appreciate your comment. We modified and added the following statement regarding the consent letter. The informed written consent letter was obtained from the parents or guardians before enrollment.

4. Your ethics statement should only appear in the Methods section of your manuscript. If your ethics statement is written in any section besides the Methods, please delete it from any other section.

We appreciate your comment. We deleted it.

5. Please include captions for your Supporting Information files at the end of your manuscript, and update any in-text citations to match accordingly. Please see our Supporting Information guidelines for more information: http://journals.plos.org/plosone/s/supporting-information.

We modified it.

Reviewers' comments:

Reviewer #1:

Despite the study was conducted in a pediatric population, this was not mentioned either in the title or in the conclusion of the manuscript. The title has to clearly state that this is a pediatric study, to attract the correct audience. In addition, the conclusion should be based only on the study results, so it has to mention the pediatric population in any drawn conclusion.

We appreciate your comment. We modified and added the following statement.

Title: The effect of short-course point-of-care echocardiography training on the performance of medical interns in children.

Conclusion: This study showed that a short teaching course could help medical interns to assess LVEF, IVCCI, and PEff in children. Therefore, it seems that adding this course to medical inerns' curricula can be promising.

All over the study, the authors exchange between medical students and medical interns. It is better to stick to the description of medical interns as they are obviously different from medical students.

We appreciate your comment. We modified it and mentioned medical interns in all sections related to our study.

The overall language of the manuscript needs a thorough revision, as some sentences are not clear and difficult to understand and other have grammatical errors. Some examples are mentioned below:
Abstract
The first sentence of the abstract is not very clear and the second one is very lengthy and difficult to understand.

We appreciate your comment. We modified it. Point-of-care ultrasound (POCUS) can add complementary information to physical examination. Despite its development in several medical specialties, there is a lack of similar studies on children by medical interns and cardiologists. Therefore, investigators aimed to assess the effect of short-course training on the performance of medical interns in point-of-care echocardiography in children.

The second sentence of the abstract results better to be rephrased for example to start with “A good agreement in terms of ... and a good agreement of ... were noted”

We appreciate your comment. We modified it as below.

A good agreement in terms of ICC and CCC for LVEF (0.832 and 0.831, respectively) and a good agreement in terms of ICC and CCC for IVCCI (0.878 and 0.877, respectively) were noted.

Keywords: could be more specific and contain the words POCUS and FoCUS

We appreciate your comment. We searched MESH and unfortunately we could not find POCUS or FoCUS. Therefore, we added Ultrasonography and Diagnostic Imaging.

Background
- “… to obtain results from the physical examination”, What does that mean? Do you mean to add data to the physical examination?

We appreciate your comment. We modified it.

**Point-of-care ultrasound (POCUS) is the application of ultrasound by non-radiologists that can add complementary, necessary, and rapid information to physical examination**

- “According to the lack of access”. I think you mean due to lack of access.

We appreciate your comment. We modified it.

- “…in referral educational and care centers”, needs further explanation

We appreciate your comment. We modified it as **referral teaching hospitals**

- “Regarding their persistent …”, you can change to “because of …”

We appreciate your comment. We modified it.

- “To now, limited studies have been performed to assess the agreement of measuring cardiac parameters by medical residents and clinicians on adults”, needs rephrasing

We appreciate your comment. We modified it as below.

**To now, limited studies on adults measured the agreement of measuring cardiac parameters by medical residents and clinicians**

- “The development of POCUS in several medical curricula focusing on its use by medical students, the rapid development of incorporating it in medicine, and the lack of similar study in pediatrics assessing the effect of limited training on medical interns”. The sentence is lengthy and unclear.

We appreciate your comment. We modified it as below.

**Despite the development of POCUS in several medical curricula ^11-12^ focusing on its use by medical interns, there is a lack of similar studies on children.**

Methods
- “short and long axes” , add “ views

We appreciate your comment. We modified it.

- “two centimeters of the junction from the right atrium”, should be “from the junction”

We appreciate your comment. We modified it as below.

From the junction of the right atrium

- Please add more details about the device model and type of the probe used.

We appreciate your comment. We modified it as below.

Ultrasound device (Samsung EKO7) with phase array probe

- “As this study aimed to assess the agreement …”. Please add the agreement between whom

We appreciate your comment. We modified it as below.

As this study aimed to assess the agreement between medical interns and the cardiologist.

Results
- “indicated LVEF >50%”, change to” indicated that LVEF was >50%”

We appreciate your comment. We modified it.

- “most patients”, should be “most of the patients”

We appreciate your comment. We modified it.

- “was considered almost perfect”, use “to be”

We appreciate your comment. We modified it.

Discussion
- “and some medical centers have already used it routinely”, the sentence needs a reference

We appreciate your comment. We added following statement and references.

FoCUS can add complementary information to routine physical examination. It is a known concept for emergency medicine specialists.

Farsi D, Hajsadeghi S, Hajighanbari MJ, Mofidi M, Hafezimoghadam P, Rezai M, Mahshidfar B, Abiri S, Abbasi S. Focused cardiac ultrasound (FOCUS) by emergency medicine residents in patients with suspected cardiovascular diseases. J Ultrasound. 2017 May 2;20(2):133-138. doi: 10.1007/s40477-017-0246-5. PMID: 28593003; PMCID: PMC5440337.

Biais M, Carrié C, Delaunay F, Morel N, Revel P, Janvier G. Evaluation of a new pocket echoscopic device for focused cardiac ultrasonography in an emergency setting. Crit Care. 2012 May 14;16(3):R82. doi: 10.1186/cc11340. PMID: 22583539; PMCID: PMC3580625.

Albaroudi B, Haddad M, Albaroudi O, Abdel-Rahman ME, Jarman R, Harris T. Assessing left ventricular systolic function by emergency physician using point of care echocardiography compared to expert: systematic review and meta-analysis. Eur J Emerg Med. 2022 Feb 1;29(1):18-32. doi: 10.1097/MEJ.0000000000000866. PMID: 34406134; PMCID: PMC8691376.

- “Based on the importance of this issue, this study showed that a limited teaching course could help medical interns to perform FoCUS for assessing LVEF”. The sentence structure needs to be changed

We appreciate your comment. We modified it as below.

Besides, this study showed that a limited teaching course could help medical interns to perform FoCUS for assessing LVEF, IVCCI, and PEff in children.

- “Results showed that the ability of medical interns was the same with residents and clinicians with higher educational levels”. Which results are you referring to here?

We appreciate your comment. We modified it as below.

Considering the agreement levels, it seems that the ability of our medical interns was the same with individuals with higher educational levels in the previous investigations 3,9

Reviewer #2:

Major issues
1) Recent studies has already assessed the efficacy of a point-of-care transthoracic echocardiography training for medical students (please see J Cardiothorac Vasc Anesth. 2021 Mar;35(3):826-833; Pilot Feasibility Stud. 2021 Sep 14;7(1):175; Postgrad Med J. 2021 Jan;97(1143):10-15. ). The authors do not mention these studies. Therefore, they should evaluate the novelty of the present study in the light of the previous ones.

We appreciate your comment. We modified it and added the above mentioned studies.

2) The manuscript requires a comprehensive editing in order to better discuss the results. The reader sometimes has the impression of reading a preliminary report without adequate elaboration.

We appreciate your comment. But as you know, this study aimed to assess the concordance between the cardiologist and medical interns regarding the three mentioned parameters and most of our results primarily reported as descriptive statistics. Although we tried to discuss our results clinically and changed some sections due to your valuable comments, unfortunately the nature of our results did not let us elaborate more in some sections.

3) In the current study, the authors claims that there was an excellent agreement (94.4%) between the cardiologist and trainees regarding LVEF. The percentage of LVEF was calculated by Teichholz formula. However, the authors should show data regarding LV end-diastolic and end-systolic internal diameters, and heart rate. Moreover, data on intraventricular wall thickness, and left ventricular posterior wall thickness at the end of systole and diastole should be provided. What about the rhythm? Please add more information.

We appreciate your great comment, but we have some ethical limitations. As you know, this preliminary study was performed by medical interns on children and indicated promising results. Despite thorough assessment of the cardiologist on each patient considering all items mentioned above, our medical interns only indicated LV end-diastolic and end-systolic diameters and our device automatically calculated LVEF by Teichholz formula. Therefore, we do not have an access to the detailed data. Besides, maybe it would be unethical to perform such a comprehensive assessment by a medical intern on a child. Certainly, a similar study including your items by individuals with higher level of education can be very helpful. We added your valuable comment as our recommendation for further studies.
