# Supplementary material for: The effect of short-course point-of-care echocardiography training on the performance of medical interns in children
Source: PLoS One. 2022 Dec 15;17(12):e0278173. doi: 10.1371/journal.pone.0278173 (PMC9754179; doi:10.1371/journal.pone.0278173)
Supplement: Attachment — Submitted filename: Response to Reviewers.docx [file pone.0278173.s005.docx]

Dear editor in chief,

We appreciate our first reviewer regarding his acceptance on our revised manuscript. Also, we acknowledge our second reviewer for his great concern. In response to his/her statement, we rechecked her/his previous comments and you can find our revisions as below.

Review Comments to the Author

Please use the space provided to explain your answers to the questions above. You may also include additional comments for the author, including concerns about dual publication, research ethics, or publication ethics. (Please upload your review as an attachment if it exceeds 20,000 characters)

Reviewer #1: Many thanks to the authors for addressing the comments
I think the manuscript is ready for publication.
Good luck!

We appreciate it.

Reviewer #2: Unfortuntaley, my questions remain unsolved. This reviewer considers these issues relevant to support the conclusions.
Regarding data, you should have collected the data at the time of the analysis. I'm really surprised that you didn't ask the question at the time of the patient evaluation.

We appreciate your comment. We put your previous comments and our responses below.

Reviewer #2:

Major issues
1) Recent studies has already assessed the efficacy of a point-of-care transthoracic echocardiography training for medical students (please see J Cardiothorac Vasc Anesth. 2021 Mar;35(3):826-833; Pilot Feasibility Stud. 2021 Sep 14;7(1):175; Postgrad Med J. 2021 Jan;97(1143):10-15. ). The authors do not mention these studies. Therefore, they should evaluate the novelty of the present study in the light of the previous ones.

We appreciate your comment. We modified it and added the above mentioned studies in our references.

**The main novelty of our manuscript can be noted as follows: Despite the development of POCUS in several medical curricula focusing on its use by medical interns, there is a lack of similar studies on children. Therefore, we aimed to measure LVEF, IVCCI, and the presence of PEff through the application of FoCUS. As we could not assess children in critical situations and do multiple FoCUS exams on one patient (child) due to ethical considerations, this study investigated the effect of short-course training on the performance of medical interns in FoCUS for measuring the parameters mentioned above in a referral pediatric cardiology ward.**

2) The manuscript requires a comprehensive editing in order to better discuss the results. The reader sometimes has the impression of reading a preliminary report without adequate elaboration.

We appreciate your comment. But as you know, this study aimed to assess the concordance between the cardiologist and medical interns regarding the three mentioned parameters and most of our results primarily reported as descriptive statistics. We tried to discuss our results clinically and changed some sections due to your valuable comments.

3) In the current study, the authors claims that there was an excellent agreement (94.4%) between the cardiologist and trainees regarding LVEF. The percentage of LVEF was calculated by Teichholz formula. However, the authors should show data regarding LV end-diastolic and end-systolic internal diameters, and heart rate. Moreover, data on intraventricular wall thickness, and left ventricular posterior wall thickness at the end of systole and diastole should be provided. What about the rhythm? Please add more information.

We appreciate your great comment, but we have some ethical limitations. As you know, this preliminary study was performed by medical interns on children and indicated promising results. Despite thorough assessment of the cardiologist on each patient considering all items mentioned above, our medical interns only indicated LV end-diastolic and end-systolic diameters and our device automatically calculated LVEF by Teichholz formula. Therefore, we do not have an access to the detailed data. Besides, maybe it would be unethical to perform such a comprehensive assessment by a medical intern on a child. Certainly, a similar study including your items by individuals with higher level of education can be very helpful. We added your valuable comment as our recommendation for further studies.
